# HDL Dysfunctionality: Clinical Relevance of Quality Rather Than Quantity

**DOI:** 10.3390/biomedicines9070729

**Published:** 2021-06-25

**Authors:** Arianna Bonizzi, Gabriele Piuri, Fabio Corsi, Roberta Cazzola, Serena Mazzucchelli

**Affiliations:** 1 Department of Biomedical and Clinical Sciences “L. Sacco”, Università di Milano, 20157 Milan, Italy; arianna.bonizzi@unimi.it (A.B.); gabriele.piuri@me.com (G.P.); fabio.corsi@icsmaugeri.it (F.C.); serena.mazzucchelli@unimi.it (S.M.); 2 Istituti Clinici Scientifici Maugeri IRCCS, 27100 Pavia, Italy

**Keywords:** high-density lipoproteins, HDL cholesterol, dysfunctional HDL, obesity, diabetes mellitus type 2, cardiovascular disease

## Abstract

High-density lipoproteins (HDLs) represent a class of lipoproteins very heterogeneous in structure, composition, and biological functions, which carry out reverse cholesterol transport, antioxidant, anti-inflammatory, antithrombotic, and vasodilator actions. Despite the evidence suggesting a clear inverse relationship between HDL cholesterol (HDL-c) concentration and the risk for cardiovascular disease, plasma HDL cholesterol levels do not predict the functionality and composition of HDLs. The importance of defining both the amount of cholesterol transported and lipoprotein functionality has recently been highlighted. Indeed, different clinical conditions such as obesity, diabetes mellitus type 2 (T2DM), and cardiovascular disease (CVD) can alter the HDL functionality, converting normal HDLs into dysfunctional ones, undergoing structural changes, and exhibiting proinflammatory, pro-oxidant, prothrombotic, and proapoptotic properties. The aim of the current review is to summarize the actual knowledge concerning the physical–chemical alteration of HDLs related to their functions, which have been found to be relevant in several pathological conditions associated with systemic inflammation and oxidative stress.

## 1. Introduction

High-density lipoproteins are one of the five major lipoproteins: chylomicrons, very low, intermediate, low, and high-density lipoproteins (VLDLs, IDLs, LDLs, and HDLs) classified by density [1]. In the basal state, HDL’s functional properties, including antioxidant, anti-inflammatory, antiapoptotic, antithrombotic effects, contribute to its antiatherogenic role [2]. However, more recent evidence has shown that oxidative stress and inflammation may lead to structural and functional HDL modifications, resulting in its conversion into a proatherogenic equivalent, despite plasma levels of HDL cholesterol [2,3].

This narrative review resumes the current knowledge concerning the relationships between physical–chemical properties of HDLs and those alterations found in the main pathological conditions characterized by systemic inflammation and oxidative stress such as obesity, T2DM, and CVD.

## 2. HDLs

HDLs are a heterogeneous and complex class of lipoproteins with densities ranging from 1.063 to 1.210 g/mL and considerable differences in size, shape, composition, and function [3,4,5,6]. In human plasma, the large, less dense (1.063–1.125 g/mL), lipid-enriched HDL2 and the small, dense (1.125–1.210 g/mL), protein-enriched HDL3 represent the two major classes of HDLs. Furthermore, these fractions can be divided into five subclasses: HDL2 and 2b, HDL3a, 3b, and 3c, according to their size [2,4,6].

HDLs consist of an apolar central core, rich in triglycerides (TGs) and cholesterol esters (CEs), surrounded by an envelope formed by amphipathic molecules such as phospholipids (PLs), free cholesterol (FC), and apolipoproteins (Apos), which act as structural elements, enzyme cofactors and ligands [1,2,3].

The main HDL protein is Apo A-I, which constitutes 70% of the protein component of HDL, while the Apo A-II represents the 15–20% and the remaining consists of other apolipoproteins also contributing to the composition of HDL, such as, Apo-IV, Apo C-I, Apo C-II, Apo C-III, Apo C-IV, Apo D, Apo E, Apo F, Apo H, Apo J, Apo L-I, Apo M, and of other enzymes such as lecithin–cholesterol acyltransferase (LCAT), cholesterol ester transfer protein (CETP) and phospholipid transfer protein (PLTP), that have a crucial a role in HDL maturation. In addition, other HDL-associated enzymes with a pivotal role in determining the HDL biological functions are the paraoxonase-1 (PON) and the platelet-activating factor acetylhydrolase (PAF-AH), which degrades platelet-activating factor (PAF), with antioxidant and anti-inflammatory activities [4,5,7].

Proteins are generally considered the main functional components of HDL, although lipids, which constitute about half of the total mass of these particles, also modulate the function of lipoproteins, affecting their metabolism or the activity of related enzymes. PLs, FC, CEs, and TGs constitute the fundamental lipid component of the HDL [4]. Cholesterol is the most measured lipid of HDL because of its potential role as an independent negative risk factor for CVD. Nevertheless, the most represented lipids in HDL are PLs (40–60 wt.% of total lipid), of which glycerophospholipids account for 35–50 wt.% and sphingolipids for 5–10 wt.% of the total HDL lipid [8]. Phosphatidylcholine (PC) and sphingomyelin (SM) are the main glycerophospholipids and sphingolipids, respectively. They regulate HDL functions and are precursors for a variety of regulatory molecules, including lysophospholipids and ceramide. In addition, sphingosine 1-phosphate (S1P), which is one of the most studied lysophospholipid signaling among those produced by the sphingolipid metabolism of the membrane, is transported bound to HDL Apo in circulatory and interstitial fluids. HDL–S1P complex activates S1P receptors on endothelial cells to suppress inflammation, and its signaling is known to be important in neuroinflammation, multiple sclerosis, and CVD [9,10,11,12,13].

Cholesterol represents 5–10 wt.% of lipids, while other sterols, such as oxysterols (27-hydroxycholesterol, 24-hydroxycholesterol, cholesterol-5,6-β-epoxide, and 7-chetocholesterol) [14], estrogens (mostly present as esters) [15], and phytosterols are found at much lower levels. TGs represent 5–12 wt.% of HDL lipids. HDL also carries vitamin E and some carotenoids, especially the polar ones, as lutein and cryptoxanthin. Vitamin E is the main lipid-soluble antioxidant in the body [16] and even if it is inserted between surface phospholipids, it can protect from oxidation both core and surface HDL lipids [17]. Lutein is a non-provitamin A carotenoid taken up preferentially into many areas of the eye, but particularly concentrated in the central region of the retina, referred to as the macula, and is the predominant carotenoid in human brain tissue [18]. Lastly, cryptoxanthin is one of the most common provitamin A carotenoids found in human blood and tissues [19].

The lipid composition affects the physical properties of either hydrophobic core or hydrophilic surface envelope of HDL, in particular their fluidity. As in cell membranes, the fluidity of the surface envelope is determined by the ratios between SM and PC, FC and PC, and total fatty acids and polyunsaturated fatty acids, since the higher are these ratios, the lower is fluidity [20,21]. The fluidity of the hydrophobic core is mainly determined by the ratio between CE and TG because the enrichment in TG increases core fluidity [21]. The state of lipid fluidity influences several HDL functions, such as cholesterol efflux from cells to HDL, the lipid exchange between HDL and other lipoproteins [22], and HDL oxidizability [21]. Finally, lipid composition can affect the conformation of HDL proteins and the activity of HDL enzymes. In particular, the ratio between CE and TG in the HDL core influences the conformation of Apo A-I [23,24], while SM is a physiological inhibitor of LCAT [25].

### HDL Function

HDLs possess several potential protective functions, as demonstrated by numerous experimental data [2,5,26,27,28,29]. A fundamental role exerted by HDLs is regarding their involvement in reverse cholesterol transport (RCT) [3]. In fact, HDLs remove the excess of cholesterol from peripheral cells, preventing its potential toxic accumulation, and transports it to the liver for bile excretion. This mechanism plays a central role in the prevention of atherosclerosis since it lets the cholesterol be efficiently removed from artery walls, where it tends to accumulate [2]. There are several steps involved in the RCT. The first stage of the RCT consists of the production of Apo A-I by the intestine and the liver, and their release into the plasma. These lipid-poor particles collect cholesterol and PL released from peripheral cells by passive diffusion (aqueous diffusion pathway and the facilitated diffusion scavenger receptor class B type 1 (SR-BI)-mediated pathway) or by the action of the ATP-dependent transmembrane transporter A1 (ABCA1) to form discoidal nascent HDL. During the second step, FC is then esterified by the LCAT enzyme and stored in discoidal HDL to form mature spherical HDL, with a lipid core enriched in CE. In addition, the ATP-binding cassette transporter G-1 (ABCG1) is another mechanism through which HDLs can acquire more cholesterol [7,30,31,32,33]. Smaller HDLs are the main cholesterol esterification site. LCAT activity transforms small and dense HDL3 into the large and light HDL2 richest in total cholesterol. The higher ratio of PC to SM in HDL3 promotes LCAT activity [22]. It is worth remembering that routine clinical measurement of HDL-c mainly reflects the levels of large cholesterol-rich HDL. To complete the RCT, a third and final step is required, during which CEs are delivered to the liver via two distinct pathways: direct and indirect. In the first case, the CEs can be removed via hepatic SR-BI. The ability of HDLs to interact with SR-BI is mainly determined by the properties of their surface lipids since it is proportional to the amount of PLs contained in HDL. Moreover, it is strongly influenced by the degree of fluidity of surface lipids [34] because cholesterol efflux from HDLs increases as the fluidity of HDL surface lipids increases [35,36]. In the second pathway, the CETP enzyme mediates the exchange between CEs and TGs from CE-rich HDLs in favor of Apo B-containing lipoproteins, VLDLs, and LDLs. Finally, these lipoproteins are rapidly captured by the liver through specific receptors and removed from the circulation by renal catabolism [3,5,7,30,31,32]. RCT exerts also an atheroprotective action because it mobilizes cholesterol from macrophage foam cells.

Moreover, several other protective mechanisms of HDL are emerging, including anti-inflammatory, antioxidant, antithrombotic, antiapoptotic, properties as well as the ability to support endothelial physiology [2,3,26,29,30,37,38]. Regarding anti-inflammatory properties, HDLs are able to inhibit the endothelial expression of adhesion molecules in response to inflammation, and to arrest the monocyte migration into the vascular wall, thus controlling the inflammatory response [5,7]. These anti-inflammatory effects are supported and enhanced by HDLs’ antioxidant functions, which are exploited to prevent LDL oxidation. Both the protein and lipid components can either be subjected to peroxidation or affect the antioxidant activity of lipoproteins. Lipoprotein peroxidation is a process characterized by chains of degrative reactions that modify both lipid and protein moieties with the formation of lipid and protein radicals. These radicals propagate the peroxidation processes and determine the accumulation of peroxidation products, mainly lipid hydroperoxides (LOOHs), reactive aldehydes, and F2-isoprostanes. HDLs constitute a major transport vehicle of LOOHs [39] and F2-isoprostanes [40] in human plasma and thus, as a consequence, prevent the accumulation of peroxidation products in LDLs. Oxidized lipids can be removed from HDLs by hepatic SR-BI [41]; hence, HDLs significantly contribute to the removal of these toxic substances from the body. The lipid composition of HDLs impacts their antioxidant activity primarily by modulation of HDLs’ physical properties. In fact, the transfer efficiency of PL hydroperoxides from LDLs to HDLs is strongly dependent on the fluidity of the surface phospholipids [42].

As regards HDL protein, Apo A-I has relevant antioxidant activity [43], as well as the HDL-associated enzymes PON1 and PAF-AH that can hydrolyze oxidized lipids and prevent LDL oxidation to protect endothelial cells [7,8,44]. Since oxidative stress and inflammation are two highly interrelated processes, the antioxidative action of HDLs contributes also to the anti-inflammatory behavior of these lipoproteins.

Moreover, HDLs have been demonstrated to promote antithrombotic activity because of their effects on the prostacyclin signaling pathway, as prostacyclins interact with nitric oxide (NO) to prevent platelet activation and aggregation. HDLs induce cyclooxygenase-2 expression, which significantly contributes to prostacyclin synthesis in endothelial cells [2,7,30]. Furthermore, it is already known that HDLs prevent endothelial antiapoptotic effects by inhibiting the induction of CPP32-like protease, resulting in a decrease in the activity of tumor necrosis factor (TNF-α), which reduces endothelial cells apoptosis [2,30].

**Figure 1:**
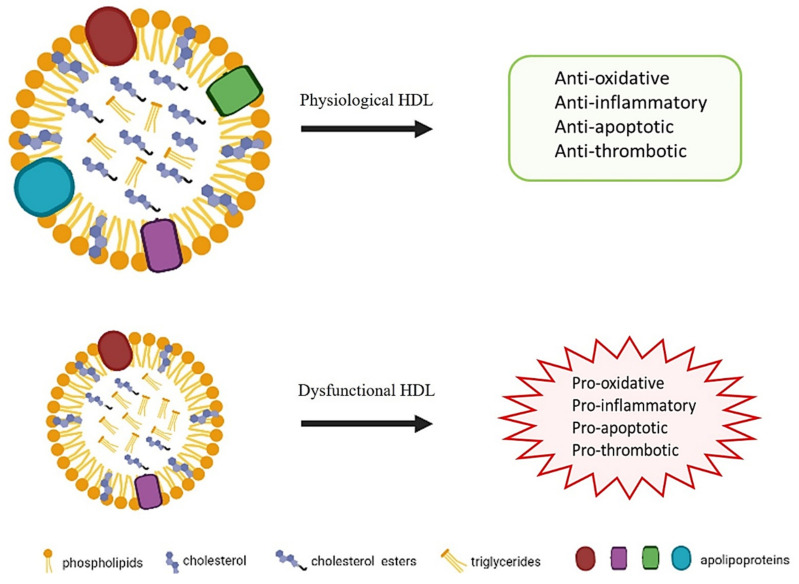
Properties of HDL in physiological and dysfunctional states.

However, emerging evidence indicates that HDL can be altered in a pathological condition, losing their atheroprotective properties [2,3,30] (Figure 1).

Recent studies are focused on the mechanisms responsible for HDL structural changes that generate the dysfunctional HDL. Dysfunctional HDL were characterized by an altered lipid and protein composition, since they show increased levels of TG, oxidized PL, an acute-phase reactants such as serum amyloid A (SAA), and decreased of Apo A-I, PON1, and PAF-AH, with negative implication on the functionality of particles [5,7,45,46].

## 3. Methods of HDL Measurement in the Laboratory

Precipitation of Apo B containing lipoproteins by using a polyanion such as heparin–MnCl_2_, phosphotungstate–MgCl_2_, dextran sulfate–MgCl_2_, or polyethylene glycol, followed by cholesterol dosage with an enzymatic colorimetric assay, constitute the standard methods to measure HDL-c. This method is fast and reproducible in clinical and specialized laboratories, despite the limitations concerning the incomplete precipitation of Apo B lipoproteins and the test conditions that influence HDL-c measurement [47]. These limitations could generate inaccurate HDL-c determinations that were found to significantly compromise the correct classification of CVD risk [48].

More refined techniques to isolate HDLs in serum include ultracentrifugation, electrophoresis, chromatography, and nuclear magnetic resonance (NMR).

Ultracentrifugation constitutes the gold standard for lipoprotein separation but is not relevant for the clinical laboratory because is expensive and technically complex [49]. High-performance liquid chromatography (HPLC) allows the simultaneous separation of lipoproteins and quantification of their cholesterol content, but their use is applied only in specialized laboratories [47]. Another method used for size separation of lipoprotein subfractions is nondenaturing polyacrylamide gradient gel electrophoresis. However, standardization between laboratories and the time required to fulfill this procedure represents the major limitations to this technique. The two-dimensional gradient gel electrophoresis is another strategy used to separate HDL. It is based on size and charge separation but remains a technique that is applied only in specialized laboratories [4,47].

Current NMR methods use proton (^1^H, ^13^C, and ^32^P) spectroscopy for the size determination and quantification of lipoprotein subclass, but it has disadvantages as well: it requires specialized equipment, and the methodology is complex to learn and perform [4,46,47,50].

The determination of HDL chemical composition is mainly achieved by using colorimetric assays [21] or more sophisticated chromatographic techniques [1,51,52,53].

Recently, modern Raman instruments coupled with microscopy and multivariate statistical analysis have made Raman spectroscopy a viable approach for the fast characterization of lipoproteins [54].

As in membranes, the fluidity of HDL lipid domains can be measured with several techniques, such as electron spin resonance, fluorescence, atomic force microscopy-based force spectroscopy, or deuterium nuclear magnetic resonance spectroscopy [55]; however, currently, none of these techniques are applicable to routine measurements.

In conclusion, unfortunately, nowadays, the techniques that allow a good qualitative–quantitative characterization of HDLs are not applicable to clinical diagnostics but could become so in the future. In our opinion, the TG dosage in HDLs and other classes of lipoproteins and the Raman spectrography are two of the most promising methods to identify possible biomarkers of lipoproteins dysfunctionality. As is currently the use of cholesterol fractions, the determination of TG concentration into the different classes of lipoproteins could provide easy-to-interpret clinical indications for identifying dysfunctional HDL [21,56]. Similarly, Raman spectroscopy could be a quick and advantageous approach for the characterization of lipoproteins that allows obtaining simultaneous information about the biomolecules composing lipoproteins: cholesterol, CE, TG, membrane lipids, unsaturated fatty acid, carotenoids, proteins, and their relative amount without any sample preparation [54]. In addition, Raman spectroscopy allows the determination of the ratio between TGs and CEs and the unsaturation degree of surface lipid fatty acids, two factors that influence the degree of fluidity of the hydrophobic core and hydrophilic surface envelope of HDLs, respectively. Hence, this analytical technique may also indirectly provide an indication of the physical properties of HDL lipids. The limitation of the application of these two approaches depends on the precipitation methods used to routinary separate the different classes of lipoproteins that interact negatively with the enzymatic dosage of triglycerides and Raman spectroscopy. It would be fascinating to overcome these technical limitations and develop a rapid method of HDL characterization [47].

## 4. Dysfunctional HDLs: From Quantity to Quality

A new viewpoint about HDL has recently emerged since HDL is no longer looked at as a protective factor or a risk factor based on the quantities of HDL-c, but as particles with many biological functions, which are not deducible only by the amount of cholesterol transported [5]. Several studies suggest that different pathological conditions can alter both the structure and function of physiological HDLs, converting these particles into dysfunctional ones [57]. These alterations are unrelated to the plasma concentration of HDL-c; thus, the quantitative measurement would not be indicative of the risk associated with HDLs. Meanwhile, structural modifications strictly related to HDLs’ functional status may be more relevant to represent their effects on the organism, debating whether could be better focus on the “quality” rather than “quantity” of HDL-c [2,3,5,50].

Data from genetic assessment and clinical trials suggest that the simple measurement of HDL-c may not be sufficient to give accurate indications about HDL functionality [57,58]. Under pathological conditions, abnormal HDLs are not simply related to the overall amount of HDL-c herein contained, but their status is a direct consequence of their overall composition. Therefore, the quality of the HDL-c appears to be a better biomarker than the quantity of HDLs [5,46,59,60]. This evidence has increased interest in studying the dysfunctional HDLs in pathological conditions characterized by systemic inflammation and oxidative stress such as obesity, T2DM, and CVD [2,5,59,61,62].

### 4.1. Obesity

Obesity is a major health problem nowadays. It is a major risk factor for both T2DM and coronary heart disease, and is strongly related to insulin resistance, hypertriglyceridemia, and reduced HDL-c level [2].

Obesity affects HDL-c levels and impacts HDL functionality, especially when associated with metabolic syndrome (MetS).

MetS includes the clustering of clinical findings of abdominal obesity, hyperglycemia, hypertriglyceridemia, low HDL-c levels, and hypertension. These risk factors could increase the prevalence of T2DM and CVD. Clinical and epidemiological studies have demonstrated that central obesity is a determining factor for MetS development [63]. Obese and MetS patients show an altered pattern of activation in several enzymes, including LCAT, CETP, PLTP; the overall amount of SAA appears to be altered as well [2,64,65].

In particular, the increased CETP activity leads to the accumulation of TGs inside the HDLs paired with the depletion of CEs and PLs [65]. As mentioned above, an increased TG-to-CE ratio changes hydrophobic core fluidity and HDL functionality. In particular, these changes in HDL lipid composition affect apolipoprotein quality and quantity, determining a decrease of Apo-AI and Apo-AII levels [66] and altered HDL functionality by decreasing HDL-induced eNOS activation [67], HDL susceptibility to oxidation [18], and their antioxidant activity [68]. Indeed, HDL metabolism is substantially altered in dyslipidemic states. Hypertriglyceridemia is associated with an increased TG-to-CE ratio in HDL core due to the action of CETP. The mechanisms leading to reduced plasma HDL cholesterol levels and HDL particle numbers in hypertriglyceridemic states are the following: (1) TG-enriched HDLs are intrinsically more unstable in the circulation, with Apo A-I loosely bound; (2) the small HDL particles, resulting from the intravascular instability of TG-enriched HDLs, are cleared more rapidly from the circulation; (3) the reduced lipoprotein lipase activity may decrease HDL levels by reducing the availability of surface constituents of TG-rich lipoproteins necessary to form nascent HDL particles [69,70]. HDLs in MetS showed alterations in composition and subfraction profile, reflecting a delay in RCT. Paradoxically, MetS patients showed higher in vitro cholesterol efflux by ABCA1 than healthy controls, which was linked to increased pre-β1-HDL and tendency to reduce LCAT mass [71,72].

Another target of modification is PON1. In obese individuals, decreased PON1 activity was accompanied by a loss of ability to inhibit lipoprotein oxidation and also correlated with increased oxidative stress [73]. Chronic inflammation typical of metabolic diseases, such as MetS and T2DM, is associated with elevated plasma levels of IL-6. As a result, the liver produces SAA, which replaces Apo A-I and PON1 in HDL. Moreover, oxidative stress, hyperglycemia, and elevated activity of CETP are other important modulators of HDL function. Oxidative stress modifies specific amino acids in Apo A-I, whereas hyperglycemia results in Apo A-I glycation. The enrichment in TGs of HDL core promotes the Apo A-I detachment from HDLs and induces conformational changes of Apo A-I that remain associated with these lipoproteins. These conformational changes make Apo A-I less accessible for the interaction with other lipoproteins, including LDLs, and reduces its ability to eliminate oxidized lipids from LDLs. Subsequent HDL hydrolysis by hepatic lipase produces small, dense HDLs enriched in TGs, serum amyloid A, and Apo A-I in an incorrect conformation that possesses deficient functionality with respect to normal HDL particles. Interestingly, weight loss promoted by both caloric restriction and bariatric surgery can improve the lipid composition of HDLs and, consequently, their functionality [56,74,75,76,77]. Despite these encouraging data, and while it is true that weight loss could reduce traditional risk factors for the development of CVD, it remains to be understood whether weight reduction also leads to a reduction in the number of events and mortality for cardiovascular pathologies in obese subjects with or without MetS. Many studies have reported conflicting results on the correlation between changes in body weight and cardiovascular risk [78,79,80,81,82]. These unexpected data need to be taken seriously in the evaluation of new cardiovascular risk assessment indices. Inflammation and oxidative stress are two of the leading features that must be considered in new studies concerning risk analysis in obese subjects. Moreover, one of the leading events, which could connect vascular oxidative stress to atherosclerosis, is the oxidative modification of lipoproteins. Indeed, oxidative damage to lipoproteins can make LDL atherogenic and reduce the antiatherogenic properties of HDL [83,84].

### 4.2. Diabetes Mellitus Type 2

T2DM is a major public health disorder characterized by inflammation and altered lipids circulation [49,85]. Patients with T2DM mostly suffer from obesity, which is why they have an increased risk for CVD. It has been suggested that low plasma HDL-c levels are associated with a higher risk of T2DM [59,86]. However, genetic studies have shown an uncertain relationship between low HDL-c levels and T2DM, directing the interest towards HDL properties. Many research studies have investigated the structural and functional alterations that can be present in dysfunctional HDLs, isolated from patients with T2DM [59,85,86,87]. There is a lot of data showing the deleterious effects of T2DM on HDL function. HDL dysfunction is mediated by several mechanisms; one of them is represented by nonenzymatic glycosylation of Apo A-I. The resulting altered apolipoprotein shows a reduced ABCA1-dependent cholesterol efflux, as well as a decrease in HDL-mediated LCAT activation. The latter one, in particular, is a key factor for RCT and for the process of esterification of cholesterol in mature HDLs [3,60,62].

Apo M is another apolipoprotein altered in this pathological condition. Apo M is reduced in T2DM patients, contributing to the proatherogenic effect of dysfunctional HDLs. In a physiological state, Apo M enhances S1P in HDLs, which results in an increased production of endothelial NO. In T2DM, the decrease of S1P promotes the proatherogenic effect, as it determines a lower production of NO [49,85]. Diabetic subjects show a decrease in NO bioavailability: such reduction of NO production leads to an inhibition of antioxidant mechanisms, which leads to an increase of oxidative stress [2,5].

In addition, in patients with T2DM, the reduction of PAF-AH and PON1 activity impairs their function as LDL antioxidative agents and, at the same time, seems to alter the antioxidant capacity of HDLs [2,49,59,62].

Apo C-III in HDL was associated with decreased insulin sensitivity even more strongly than plasma total Apo C-III and could be considered a promising target for T2DM prevention and treatment [88]. HDL lacking Apo C-III was inversely associated with the incidence of T2DM. Similarly, only HDL lacking Apo C-III was beneficially associated with plasma glucose, HbA1c, and insulin sensitivity, while higher HDL containing Apo C-III was associated with lower insulin sensitivity [88].

Moreover, the presence of chronic inflammation in T2DM increases the levels of SSA. This alteration in HDL proteome contributes to the acceleration of the clearance of these particles from plasma [5,45,49,85].

Another structural change includes the altered lipid composition of HDL, insulin deficiency, and elevated CETP activity, which leads to an increase of triglycerides, contributing to HDL dysfunction in T2DM [5,49].

### 4.3. Cardiovascular Disease

CVD is by far the leading cause of morbidity and mortality globally, and it represents a relevant problem for public health because its origin is often caused by several other clinical or subclinical conditions. For instance, hypertension, obesity, MetS, and dyslipidemia are currently considered the main risk factors in the progression of CVD [3]. For decades, a topic of great interest has been the complex relationship between HDL-c, universally regarded as the “good cholesterol”, and cardiovascular risk [3,5]. Epidemiologic studies have supported an inverse relationship between low HDL-c concentrations and high cardiovascular risk, supporting the hypothesis that a higher level of HDL-c is “better” [31,45].

However, this assumption has been recently challenged by clinical and genetic studies since many patients with normal or even high plasma HDL manifested CVD events [46,89,90]. Genetic studies in humans also revealed that specific genetic variants associated with increased HDL-c do not protect against CVD [3,5,91]. These findings raised the question about the suitability of HDL-c levels as good parameters to follow in the clinic, suggesting that the clinical relevance of changes in HDL biological function might be more useful to study if compared to HDL concentration alone [3,7,49].

The inflammation burden in CVD leads to the development of dysfunctional HDLs, resulting in changes in their protein or lipid composition as well as altered functions [5,46].

In this pathological condition, HDLs lose their atheroprotective properties and become proatherogenic. Indeed, HDLs isolated from patients with coronary heart disease cannot inhibit oxidation of LDLs, whereas, in healthy conditions, they seem to have a role in protecting LDL from oxidative stress. Furthermore, dysfunctional HDLs show an increased expression of inflammatory adhesion markers and seem to promote the production of endothelial superoxide. Such modifications contribute to creating an oxidative environment, which eventually leads to damage in LDLs as well [5,92]. In addition, HDLs from patients with CVD show an increase in SAA levels, due to the action of inflammatory cytokines, and the increase in the amount of SAA is often preceded by a decrease in the levels of circulating Apo A-I [45]. Additionally, SAA-rich HDLs lose their antioxidative potential since, in this context, reduced activity of both PON1 and PAF-AH was recorded [86,92]. Inflammatory disorders are also characterized by a considerable increase in oxidative stress. Regarding HDLs, such a condition leads to lipid peroxidation as a side effect of the oxidative conditions, impairing the characteristics of previously functional HDLs [92]. Here, Apo A-I becomes a substrate for myeloperoxidase (MPO), which is known to be a mediator of phlogosis in macrophages and other leukocytes as well, which can convert HDL to a dysfunctional form. Therefore, in this pathological context, MPO may promote oxidative damage by Apo A-I oxidation and, concurrently, it may block plaque regression, since modifications of HDL/Apo A-I appear to interfere with LCAT activation [3,45].

The 2019 ACC/AHA Guideline on the Primary Prevention of CVD suggests that to evaluate cardiovascular risk, it is essential to focus primarily on total cholesterol value and LDL cholesterol (LDL-c) value, considered the “bad cholesterol” [93]. The values of LDL-c alone acquire significance only in subjects with a higher cardiovascular risk [93]. In these cases, the therapeutic target aims to reduce LDL cholesterol levels as much as possible, even below 40 mg/dL, while in healthy subjects without particular risk factors, much higher levels are accepted (up to 116 mg/dL) [93]. The total cholesterol values considered safe have progressively lowered, but the value of total cholesterol, HDL-c, and LDL-c alone are considered insufficient to define a healthy person’s cardiovascular risk [94,95,96].

Experimental studies in mice have demonstrated that HDL-mediated macrophage cholesterol efflux capacity is causally related to atherosclerosis [97]. Findings from human studies have shown a significant inverse correlation between cholesterol efflux capacity and prevalent coronary heart disease, generally regardless of HDL cholesterol level [98,99,100,101,102]. On the other hand, elevated cholesterol efflux was also paradoxically associated with increased prospective risk for myocardial infarction, stroke, and death [101,103]. The observation that cholesterol efflux measurements are generally not correlated with HDL-c levels seems to suggest that the former do not measure the same factor that was originally discovered in epidemiological studies on HDL cholesterol levels [89]. These observations further support the need to consider in future studies the physical or compositional factors that are responsible for the ability of HDL to promote the cholesterol efflux from macrophages, as well as the elements influencing these factors.

Considering the effects of the ratio between TG and CE on lipoprotein functionality described above, it is not surprising that the association of HDL-c with triglyceridemia could increase the prognostic and diagnostic value of HDL-c. In fact, a high TG/HDL-C ratio is considered a cardiometabolic risk, especially for children, adolescents, and renal failure patients [104,105,106,107,108,109].

The association of HDL-c with inflammatory indices could also increase the prognostic and diagnostic values of HDL-c. For example, an index that is gaining interest is the relationship between monocytes and HDL-c. Accumulation of monocytes and reduction of HDL-C may participate in atherosclerosis and CVD. Given that the relationship between the high number of monocytes and low HDL-c levels has been reported in inflammatory disorders, the monocyte to HDL ratio could be a convenient marker to predict atherosclerosis development and progression [110,111,112].

Nonetheless, since the many physiological functions of HDL ultimately depend on their physical–chemical properties, it is desirable that methodologies that allow accurate characterization of these properties will soon be available and will be applicable to clinical trials.

## 5. Conclusions

HDLs have a remarkable variability of physiological functions that have been found altered in pathological conditions such as obesity, T2DM, and CVD, and in general are associated with conditions of systemic inflammation and increased oxidative stress.

Therefore, the importance to define the amount of cholesterol transported by these lipoproteins, in addition to physical–chemical parameters related to HDL functions, should be investigated as biomarkers in pathological conditions.

## Data Availability

Not applicable.
